# Data on the sustainability profile and food waste management in primary and secondary schools: The case of the Catalonia region in Spain

**DOI:** 10.1016/j.dib.2019.104825

**Published:** 2019-11-19

**Authors:** Belén Derqui, Didier Grimaldi

**Affiliations:** a Department of Business Management, IQS School of Management. Universitat Ramon Llull, Vía Augusta 390, 08017, Barcelona, Spain; b Department of Business Management. Universitat Ramon Llull, Sant Joan de La Salle, 42, 08022, Barcelona, Spain

**Keywords:** Sustainable schools, Food waste, Canteen management, Waste reduction initiatives, Cohesion policy

## Abstract

This paper presents data from a survey administered to 548 public and private school headteachers in Catalonia (Spain) in 2018 on sustainability practices and food waste management. Raw data were collected through a standardised and structured questionnaire (For more information refer to Building and managing sustainable schools: The case of food waste [1]). The variables of the dataset include items relative to the canteen management system, the school sustainability profile and perceptions on food waste generation and management. Additionally, data on the level of implementation and interest on a wide range of potential initiatives to fight against food waste are provided within this article. School-level attributes (e.g. size, infrastructure) are also included.

**Table:** Specifications Table

Subject Area	Business, Management and Accounting:
Specific subject area	Strategy and Management
Type of data	Table and graphs
How data were acquired	Data were acquired from an email survey answered by 548 school headteachers in Catalonia, Spain (420 offering canteen service). The complete questionnaire can be found in the referred article, https://doi.org/10.1016/j.jclepro.2019.118533.
Data format	Raw, calculated
Parameters for data collection	The data collection includes 4 mandatory parameters: school general and demographic data, canteen management data, school sustainability data and food waste data.
Description of Data Collection	The data were collected responding to a structured questionnaire asking headteachers to express their perception about the efforts and the initiatives realised by the school in terms of sustainability and the food waste management
Experimental factors	The raw data collected were organised in a spreadsheet. The information was organised by alphabetic order, using the name of the School as sorting criterion.
Experimental features	Raw data relative to the school profile and food waste management efforts and initiatives were used to calculate statistical indicators and visualised in different graphs.
Data source location	Catalonia (Spain)
Data accessibility	Raw data was deposited at Mendeley dataset website with the following address: https://doi.org/10.17632/c9ptkvpt25.1
Related Research Article	Derqui, B.; Grimaldi, D.; Fernandez, V; Building and managing sustainable schools: The case of food waste, Journal of Cleaner Production, Volume 243, January 2020, 118533. https://doi.org/10.1016/j.jclepro.2019.118533

**Table:** 

**Value of the Data** • These data can be used to analyse the sustainability profile of primary and secondary schools in Catalonia, Spain. • The data present an overall benchmarking between different school canteen management systems. • The dataset makes it possible to identify and evaluate school food waste management efforts based on the initiatives that are put forward. • The data can be processed by implementing a variety of statistical techniques (i.e., descriptive statistics, multivariate regression, cluster analysis). • Policy makers and other stakeholders can benefit from these data to set and assess best practices for the improvement of the sustainable performance of schools. • The data can be used to calculate new indicators on the level of execution (and interest) of initiatives aiming to minimise food waste at school canteens.

## 1 Data

Scholars have found school canteens to be a relevant source of food waste, which is a growing ethical, environmental and economic problem [2]. Collecting and analysing data on school sustainability initiatives in general - and food waste management in particular - are interesting tasks to define, measure and rank the school's sustainability performance. Furthermore, if our children learn sustainable habits at school, they will apply sustainable actions during the rest of their life and may become agents of change [3]. Nevertheless, school managers, despite making increasing efforts towards sustainability, are not yet committed to minimising food waste.

Against this background, researchers collected data from school headteachers to illustrate primary and secondary school sustainability profile and perceptions on food waste generation and management. Additionally, a broad range of potential initiatives to minimise food waste at school canteens were presented and respondents graded their interest in their implementation according to a 5-Point Likert scale: from “1: not at all” to “5: to an extreme extent”. Furthermore, if the initiative is currently implemented at their school, the respondents could answer: “Currently applied”.

**Graph 1:**
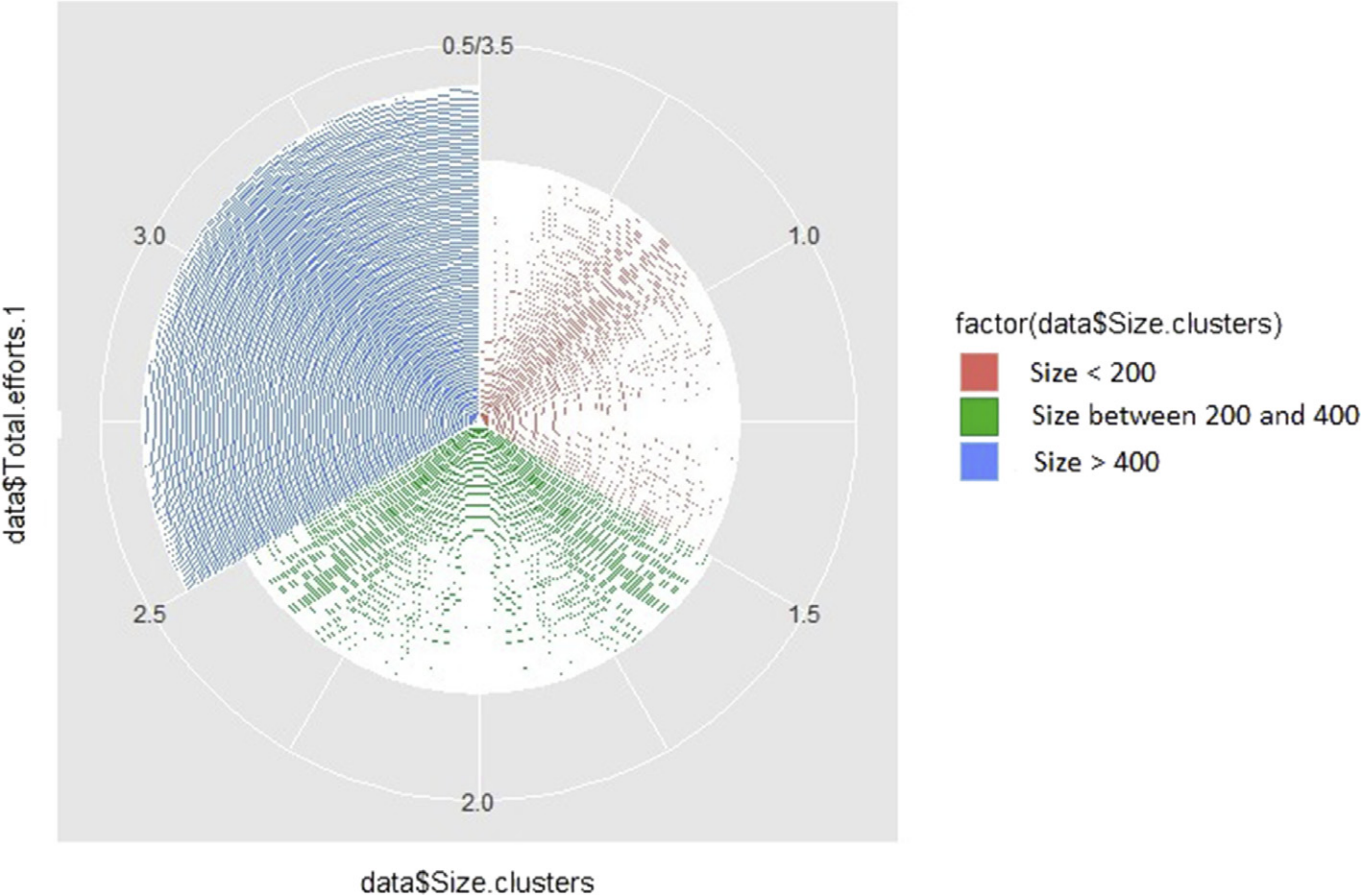
Sustainability profile by school size.

**Graph 2:**
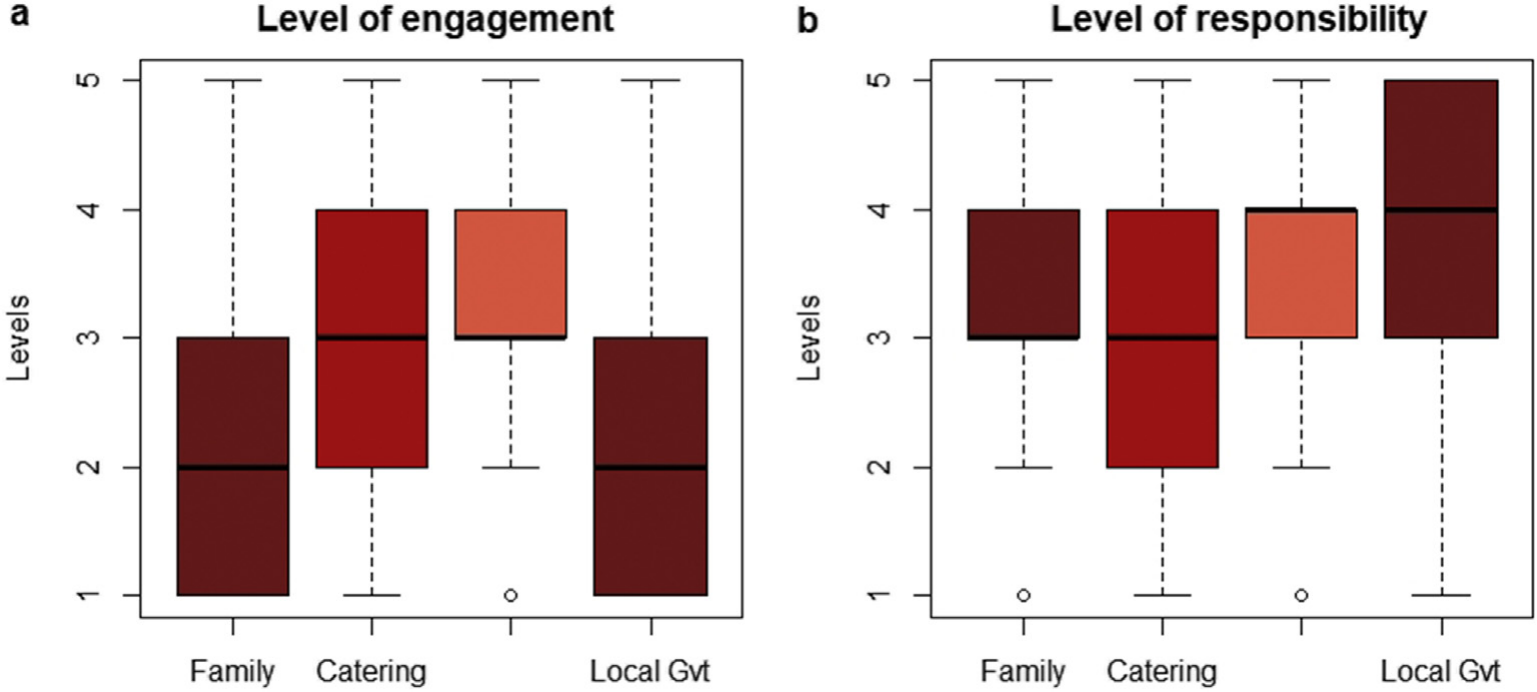
Perceived level of engagement and responsibility for food waste reduction.

**Graph 3:**
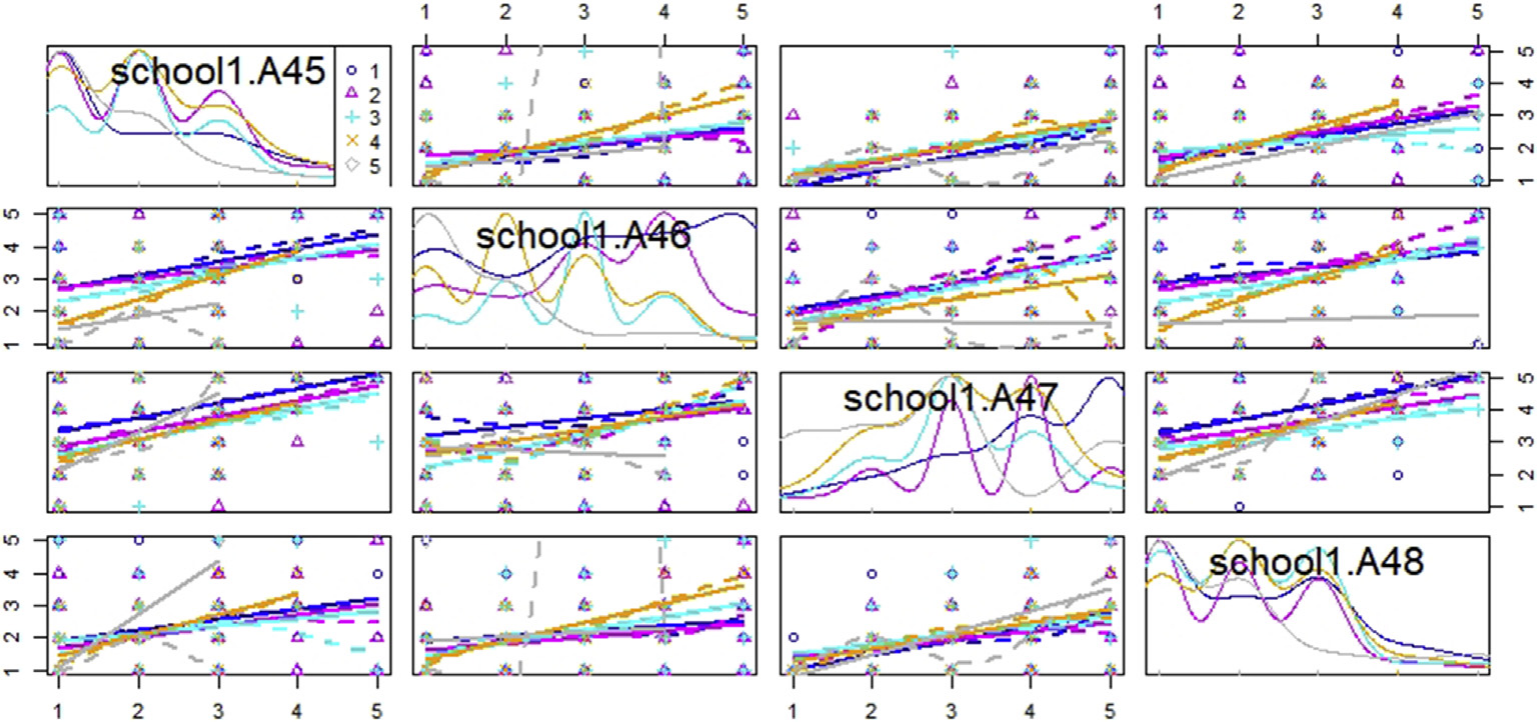
Relationship between engagement and food waste generation.

**Graph 4:**
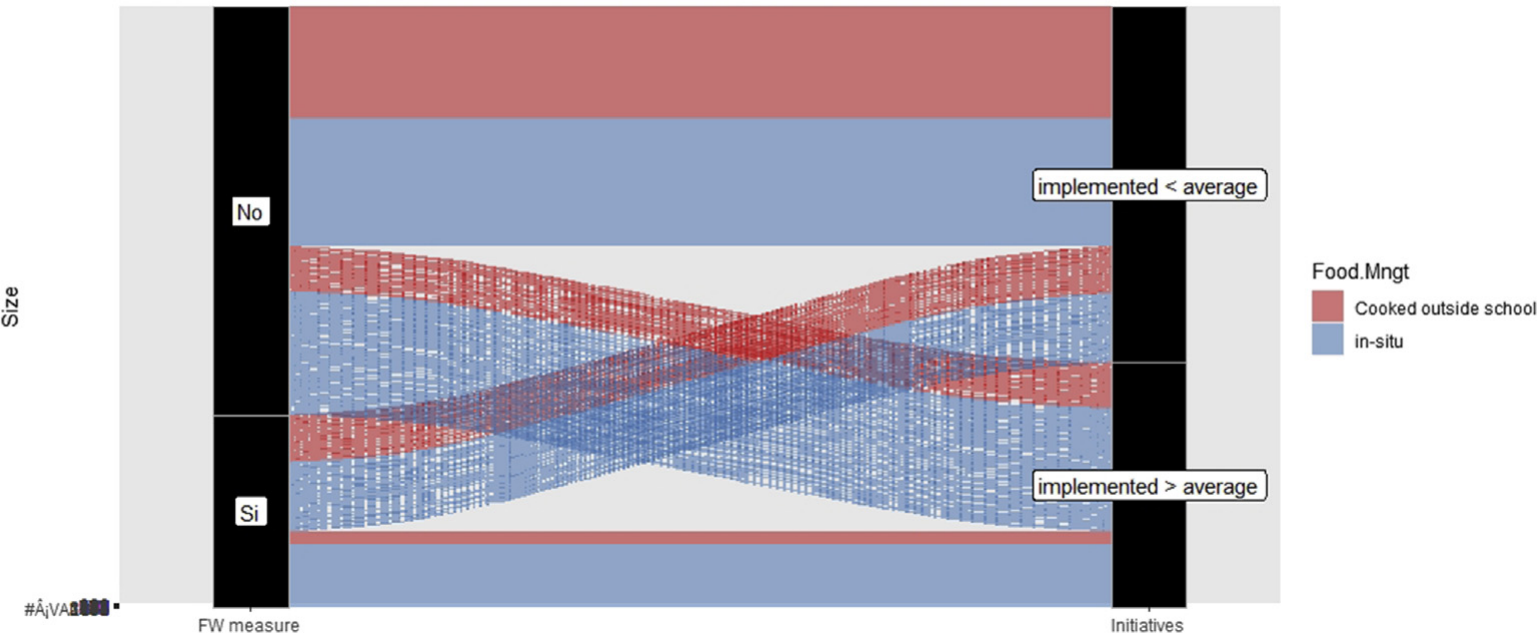
Relationship between Food Waste measurement and Execution of Initiatives.

**Graph 5:**
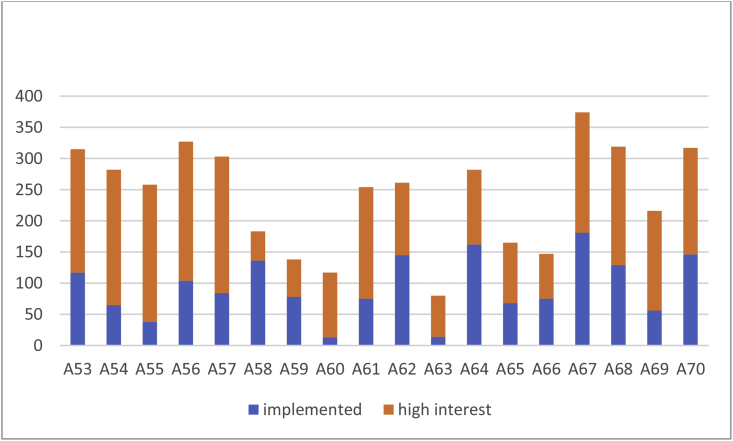
Acceptance rate of food waste reduction initiatives.

**Graph 6:**
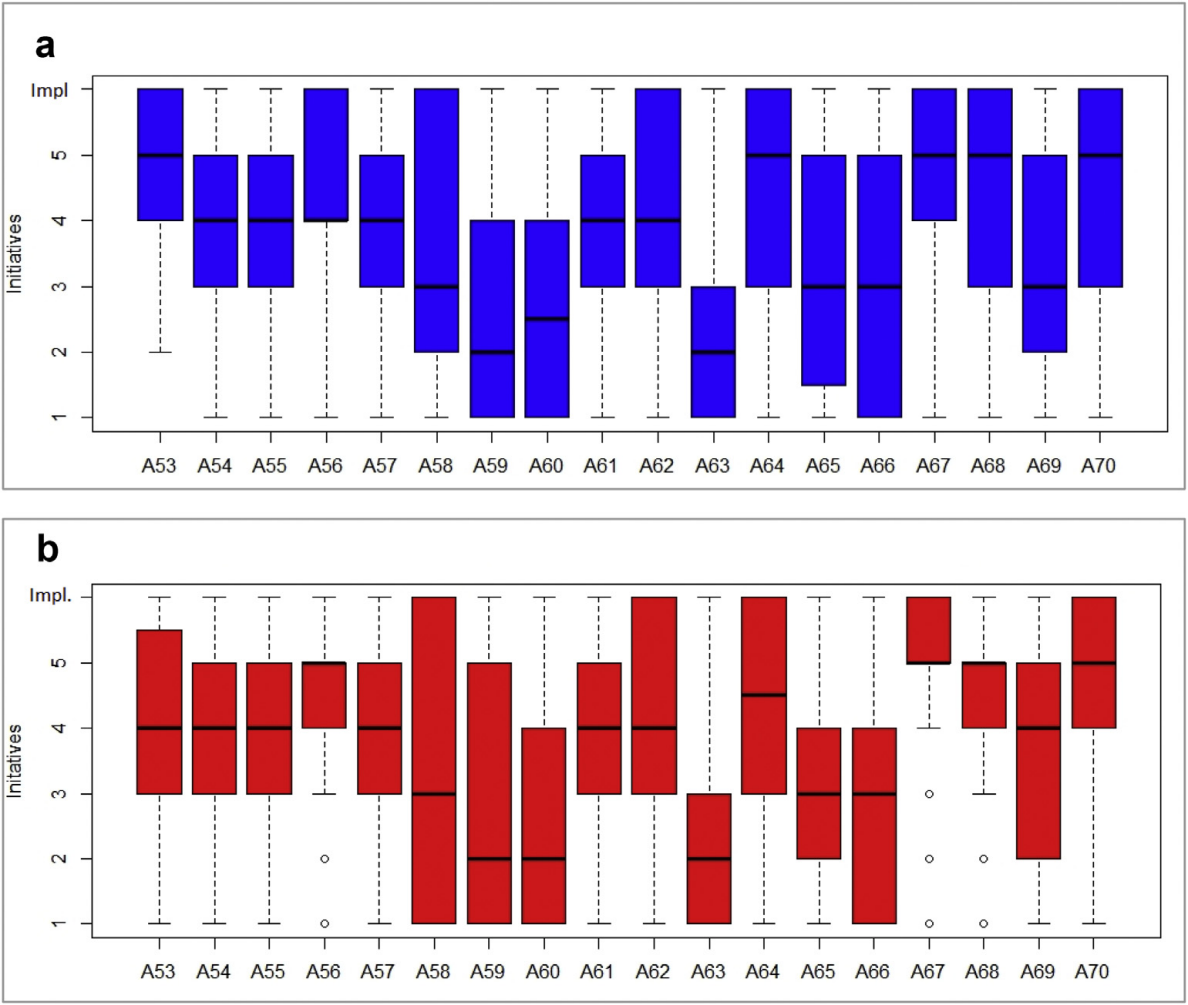
Acceptance rate of food waste reduction initiatives by school sustainability certification.

The dataset, which can be accessed at Mendeley dataset website (https://doi.org/10.17632/c9ptkvpt25.1), contains self-reported responses of individual survey participants. Table 1 shows demographic information on the sample. The graphs within the article aim to represent visually the richness of the dataset. Graph 1 shows schools’ sustainability profile by size; Graph 2, Graph 3 refer to engagement and responsibility on food waste reduction; Graph 4, Graph 5, Graph 6 refer to the responses with regard to the 18 different food waste reduction initiatives proposed. For more information about the survey questionnaire, please refer to Derqui, B.; Grimaldi, D.; Fernandez, V; Building and managing sustainable schools: The case of food waste, Journal of Cleaner Production, Volume 243, January 2020, 118533 [1]. https://doi.org/10.1016/j.jclepro.2019.118533.

**Table 1:** Sample profile (in %).

Canteen Business Model		Respondent Role at School		Size of Schools (Number of Primary Students)		Size of Schools (Number of Secondary students)
In situ kitchen	48%	Top Management	83%	Mean	175	373
Cooked outside No food service	28% 24%	Administration	11%	Up to 200	49%	30%
% Own a Sustainability Certificate: No: 66% Yes: 34%		Canteen Manager	6%	Over 400	13%	38%
				200–400	38%	32%

The target population included in the dataset consisted of Primary and Secondary Education Schools in Catalonia, Spain with and without dining services. A link to the online questionnaire was sent to 5441 schools, addressing school top management. We received 548 valid responses. A total of 83% (n = 455) respondents were Directors or Headteachers of the school; 11% (n = 50) were heads of studies or had an administrative role in the school and 6% were canteen managers, as shown in Table 1.

Most of the schools in our sample (76%) offered dining services to their students. The mean number of students per school was 175 in Primary Education and 373 in Secondary Education. While 81% of the schools in the sample were public, 19% were private.

The sum of items ES1 to ES5 and SS1 to SS4 of the questionnaire [For more information refer to 1] defines a new indicator of the sustainability profile of the schools, as shown in Graph 1. The grouped data are then weighted according to the number of answers received (to be reasonably compared) and clustered in 3 groups by the number of students at the school (below 200 students, between 200 and 400 students and over 400 students).

Graph 2a, b show which institution (families, catering companies, school management and local government) is considered to be engaged and responsible for the reduction of food waste respectively.

Graph 3 shows the relationships between the declared amount of food waste generation (A42) and the level of perceived engagement from the different school stakeholders.

Graph 4 shows the relationship between the fact that the food waste generated at the canteen is measured and initiatives undertaken to fight against it. We separate schools in two groups: those that have implemented initiatives above and below the average (4.01). Graph 4 also illustrates the relationship between the canteen management system (in-situ kitchen or food brought from outside the school) and the implementation of initiatives to minimise food waste.

In graph 5 we measure, for each of the proposed initiatives, the level of current execution (blue fill) and the interest on its implementation when the initiative is not yet being implemented (orange fill). To do this, we added the percentage of respondents stating that the initiative was currently being implemented at their schools to the percentage of those who answered they had a high interest in its implementation. We consider high interest when the answer to a Likert scale item 1–5 is 4 or 5.

Graph 6a, b, respectively, show the interest declared on each initiative differentiating whether the school owns or not a sustainability certificate. To build the Y-axis, we considered the answer “currently implemented at my school” as a 6th point, adding to the 1–5 Likert scale option.

**Graph 7:**
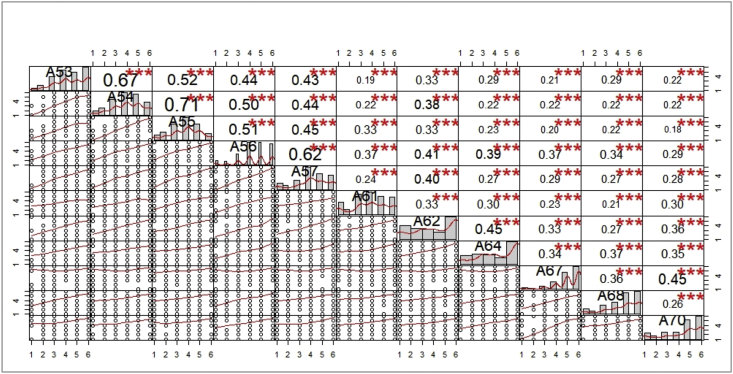
Correlation between food waste reduction initiatives.

Graph 7 computes a correlation matrix between the better-ranked initiatives among those proposed in the questionnaire to reduce food waste (S1 to S17).

## 2 Experimental design, materials and methods

### 2.1 Experimental design

The measures contained in the provided dataset were gathered from primary research and a survey questionnaire. For more information, refer to Ref. [1] The questionnaire includes items related to the Canteen Management systems and the facilities of the School. It also includes those questions related to the School Sustainability profile. Moreover, it measures Food Waste management issues. Finally, it lists 17 different potential interventions to fight against food waste in order to measure the grade of implementation and interest on each of them.

### 2.2 Data

Data are available as comma separated values (.csv). The file can be accessed via Mendeley Data.

### 2.3 Method

The data were obtained from primary and secondary schools located in the region of Catalonia, in Spain through a structured questionnaire created by the authors. Email addresses were obtained from official open access sources. Emails including a link to the survey were sent to the whole universe of school headteachers in the region in February 2016. To mitigate ethical concerns in survey research, we aimed at protecting respondents by providing full transparency on the purpose and motivation of the research and ensuring the anonymity of research participants during and after completing the survey. After several reminders and eliminating responses with missing data, the dataset come down to 548 valid responses for a yield ratio of 10.1%.
